# Supplementary material for: Fine Structure of the Mouthparts of Three Tomicus Beetles Co-Infecting Pinus yunnanensis in Southwestern China with Some Functional Comments
Source: Insects. 2023 Dec 7;14(12):933. doi: 10.3390/insects14120933 (PMC10743386; doi:10.3390/insects14120933)
Supplement: Supplementary file 1 [file insects-14-00933-s001.zip › Figure S1.pdf]

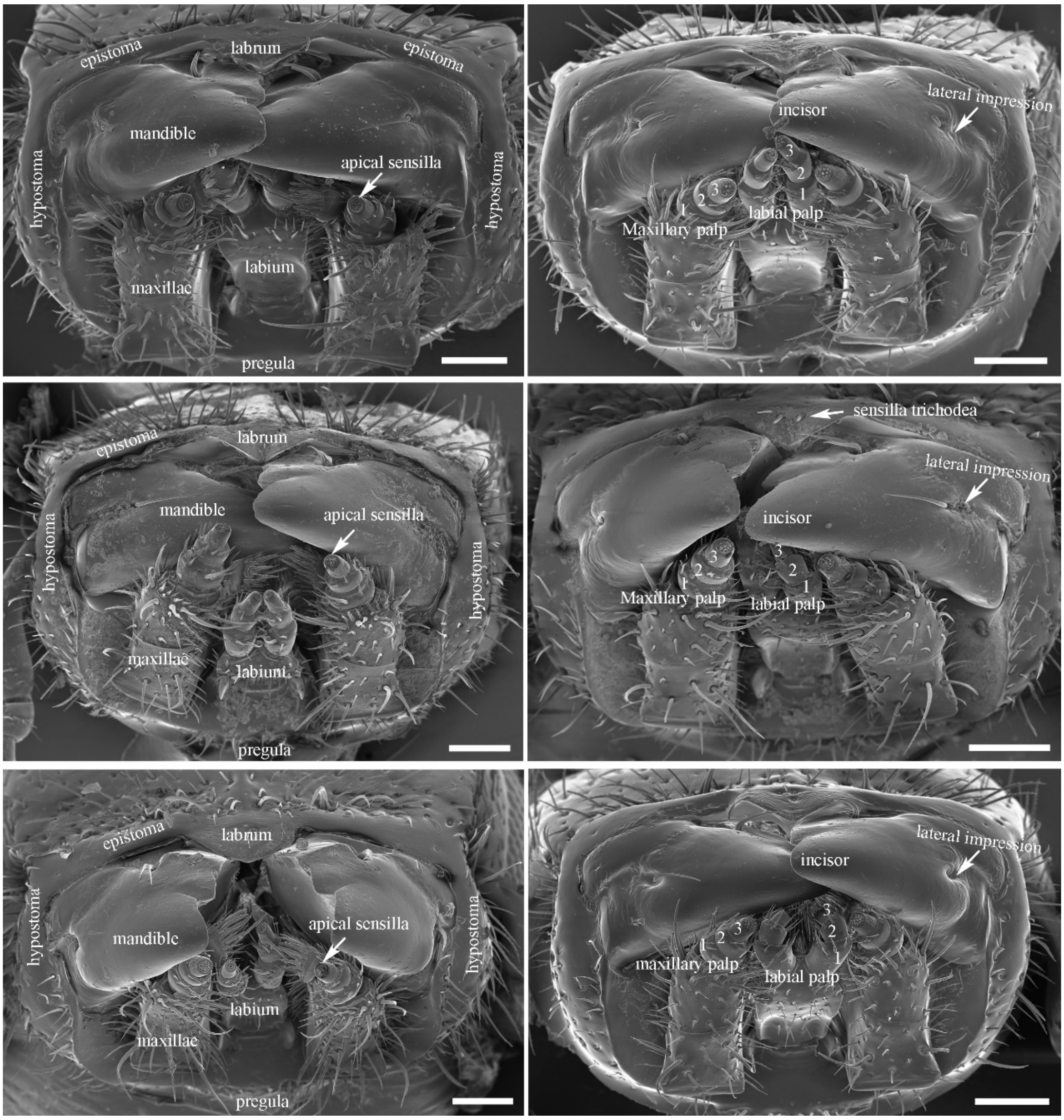

Figure S1. The overall mouthpart of a female (left) and a male (right) of three *Tomicus* beetles. Top, *T. yunnanensis*; mid, *T. brevipilosus*; bottom, *T. minor*. Scale bar = 100 μm.
