# Supplementary material for: Fine Structure of the Mouthparts of Three Tomicus Beetles Co-Infecting Pinus yunnanensis in Southwestern China with Some Functional Comments
Source: Insects. 2023 Dec 7;14(12):933. doi: 10.3390/insects14120933 (PMC10743386; doi:10.3390/insects14120933)
Supplement: Supplementary file 1 [file insects-14-00933-s001.zip › Figure S2.pdf]

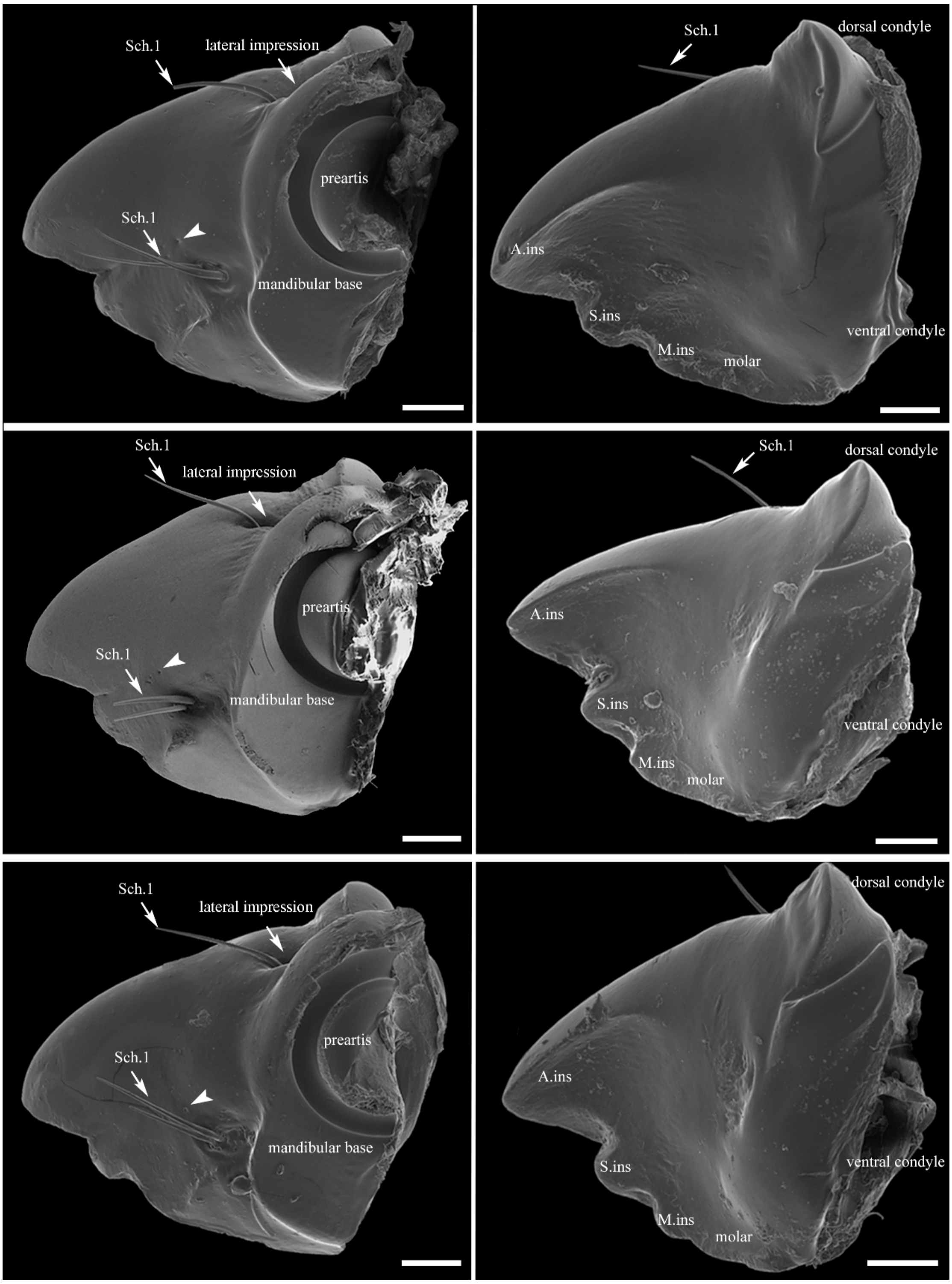


Figure S2. The mandible of an external (left) and an internal (right) view of the three *Tomicus* beetles. Top, *T. yunnanensis*; mid, *T. brevipilosus*; bottom, T. minor; Sch.1, sensilla chaetica 1; A.ins, apical incisor; S.ins, subapical incisor; M.ins, median incisor. The equilateral arrowhead shows a unique area of cuticular pores on the mandible. Scale bar = 50 μm.
