# Supplementary material for: Fine Structure of the Mouthparts of Three Tomicus Beetles Co-Infecting Pinus yunnanensis in Southwestern China with Some Functional Comments
Source: Insects. 2023 Dec 7;14(12):933. doi: 10.3390/insects14120933 (PMC10743386; doi:10.3390/insects14120933)
Supplement: Supplementary file 1 [file insects-14-00933-s001.zip › Figure S4.pdf]

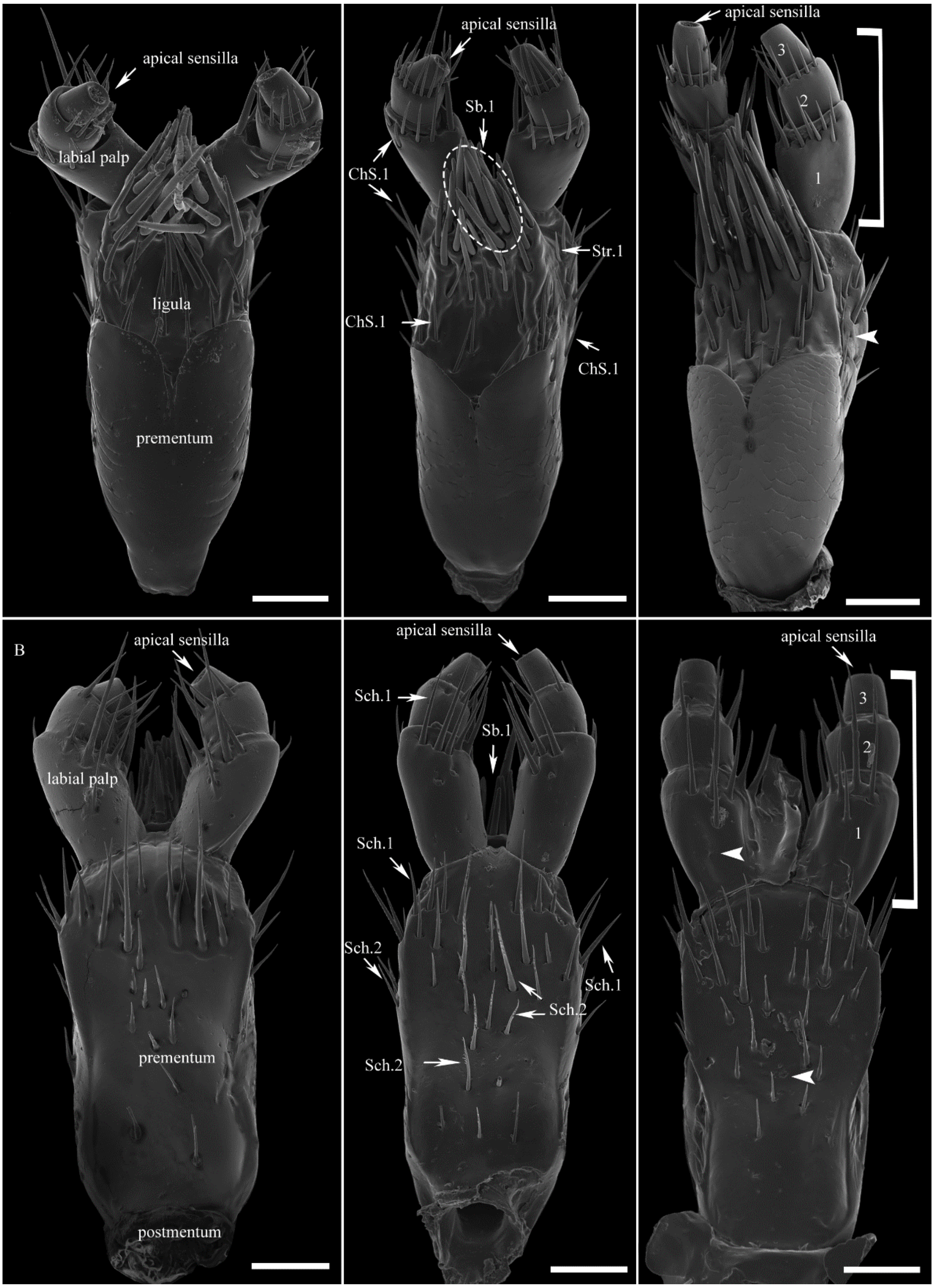

Figure S4. The labium of an external (top) and internal (bottom) view of the three *Tomicus* beetles. left, *T. yunnanensis*; mid, *T. minor*; right, *T. brevipilosus*; Sch.1-2, sensilla chaetica 1 and 2; Str.1-2, sensilla trichodea 1 and 2. The dotted line shows a common area of Sb.1. The equilateral arrowhead indicates the cuticular pores on the maxillae. Scale bar = 50 μm.
