# Supplementary material for: Fine Structure of the Mouthparts of Three Tomicus Beetles Co-Infecting Pinus yunnanensis in Southwestern China with Some Functional Comments
Source: Insects. 2023 Dec 7;14(12):933. doi: 10.3390/insects14120933 (PMC10743386; doi:10.3390/insects14120933)
Supplement: Supplementary file 1 [file insects-14-00933-s001.zip › Figure S5.pdf]

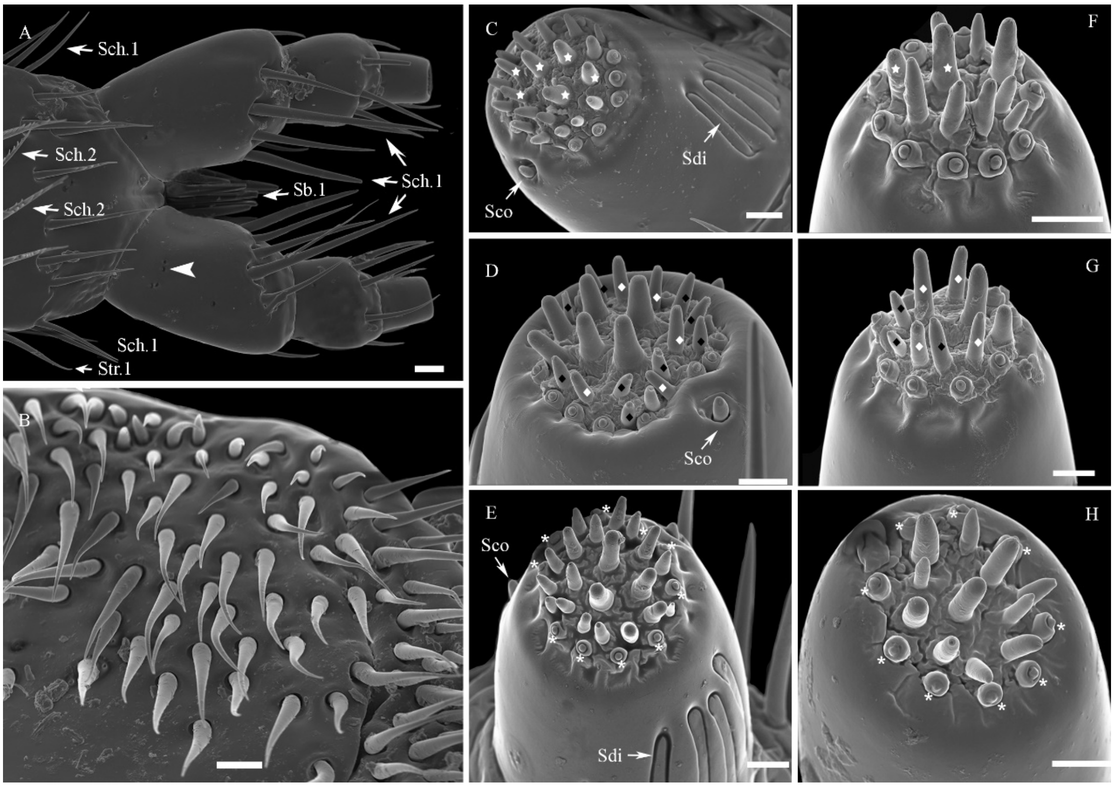

Figure S5. Sensilla type and distribution on the mouthpart elements of the three *Tomicus* beetles. (A) Several sensilla types occur on the terminal prementum of labial palp in the *T.minor*, note the difference between two Sch.2; (B) Many Str.2 concentrates on the ventral view of the galeo-lacinial complex in *T. brevipilosus*. (C-E) Apical sensilla occur on the maxillary palp of *T. yunnanensis*, *T. minor*, and *T. brevipilosus*, respectively. (F-H) Apical sensilla occur on the labial palp of *T. yunnanensis*, *T. minor*, and *T. brevipilosus*. White stars show sensilla basiconia 2; white and black diamonds show sensilla twig basiconica1 and 2, respectively; asterisks show sensilla twig basiconica 3. Sch.1-2, sensilla chaetica 1 and 2; Str.1-2; sensilla trichodea 1 and 2; Sb.1, sensilla basiconica 1; Sco, sensilla coeloconica. Scale bar, A-B = 10 μm, C-H = 5 μm.
